# Prolonged Repellent Activity of Plant Essential Oils against Dengue Vector, *Aedes aegypti*

**DOI:** 10.3390/molecules28031351

**Published:** 2023-01-31

**Authors:** Abdullah Haris, Muhammad Azeem, Muhammad Ghazanfar Abbas, Muhammad Mumtaz, Raimondas Mozūratis, Muhammad Binyameen

**Affiliations:** 1 Department of Entomology, Faculty of Agricultural Sciences and Technology, Bahauddin Zakariya University, Multan 60800, Pakistan; 2 Department of Chemistry, Abbottabad Campus, COMSATS University Islamabad, Abbottabad 22060, Pakistan; 3 Department of Zoology, Stockholm University, Svante Arrhenius väg 18B, SE-10691 Stockholm, Sweden; 4 Laboratory of Chemical and Behavioural Ecology, Institute of Ecology, Nature Research Centre, LT-08412 Vilnius, Lithuania

**Keywords:** mosquito repellent, gas chromatography-mass spectrometry, bioactive compounds, *Perovskia atriplicifolia*, *Citrus reticulata*

## Abstract

Repellents are effective personal protective means against outdoor biting mosquitoes. Repellent formulations composed of EOs are finding increased popularity among consumers. In this study, after an initial screening of 11 essential oils (EOs) at the concentration of 33 μg/cm^2^, five of the most repellent EOs, *Perovskia atriplicifolia*, *Citrus reticulata* (fruit peels), *C. reticulata* (leaves), *Mentha longifolia*, and *Dysphania ambrosioides* were further investigated for repellent activity against *Aedes aegypti* mosquitoes in time span bioassays. When tested at the concentrations of 33 μg/cm^2^, 165 μg/cm^2^ and 330 μg/cm^2^, the EO of *P. atriplicifolia* showed the longest repellent effect up to 75, 90 and 135 min, respectively, which was followed by *C. reticulata* (peels) for 60, 90 and 120 min, *M. longifolia* for 45, 60 and 90 min, and *C. reticulata* (leaves) for 30, 45 and 75 min. Notably, the EO of *P. atriplicifolia* tested at the dose of 330 μg/cm^2^ showed complete protection for 60 min which was similar to the commercial mosquito repellent DEET. Gas chromatographic-mass spectrometric analyses of the EOs revealed camphor (19.7%), limonene (92.7%), sabinene (24.9%), carvone (82.6%), and *trans*-ascaridole (38.8%) as the major constituents of *P. atriplicifolia*, *C. reticulata* (peels), *C. reticulata* (leaves), *M. longifolia*, and *D. ambrosioides*, respectively. The results of the present study could help develop plant-based commercial repellents to protect humans from dengue mosquitoes.

## 1. Introduction

The yellow fever mosquito, *Aedes aegypti* L. (Diptera: Culicidae) is a vector of 54 different viruses and 2 species of *Plasmodium* causing dengue fever, chikungunya, zika, mayaro, yellow fever, and many other diseases [1]. The geographic distribution of *Ae. aegypti* is increasing rapidly and according to one estimate, half of the world’s population lives in areas where the environment has become suitable for yellow fever mosquitoes due to the abrupt climatic changes [2].

*N*,*N*-diethyl-meta-toluamide (DEET) is considered a gold-standard mosquito repellent for the protection of people against mosquito bites [3,4]. However, several studies have reported its adverse effects such as skin reactions, encephalopathies etc. upon extended usage [5,6,7]. Considering the toxic impact of DEET to humans, several plant species have been explored with the aim of finding natural compounds that could be used as an alternative to synthetic repellents. For example, *Magnolia grandiflora* [8], *Mentha spicata* [9], *Citrus aurantifolia* [10], *Cymbopogon citratus* [11], *C. nardus* [12], *Dianthus caryophyllum* [13] *M. piperita* [14], *Nepeta cataria* [15], *Ferronia elephantum* [16] and *Carpesium abrotanoides* [17] proved to be effective as repellents against *Ae. aegypti*. Moreover, the chemical constituents of a few EOs, such as thymol, carvacrol and α-terpinene [18], β-caryophyllene oxide [19], 1,8-cineole [20] and *trans*-nerolidol [17] have also been reported for their repellent effects against different mosquito species. 

To find new plant-based sources of repellents for yellow fever mosquitoes, we have searched for plant species which showed biological activities against other insect pests but were not tested as repellents against *Ae. aegypti*. For example, *Perovskia atriplicifolia* has been reported as an antifeedant against *Tribolium castaneum* (Coleoptera: Tenebrionidae) [21] while *Eucalyptus camaldulensis* acted against *Helicoverpa armigera* (Lepidoptera: Noctuidae) [22]. Moreover, the fumigant toxicity of *E. camaldulensis* was also documented against *Callosobruchus maculatus* (Coleoptera: Chrysomelidae), *Sitophilus oryzae* (Coleoptera: Curculionidae) and *T. castaneum* [23]. The insecticidal and repellent activities of *M. longifolia*, *C. reticulata* and *Dysphania ambrosioides* were observed against *Tribolium confusum* (Coleoptera: Tenebrionidae), *C. maculatus* and *Sitophilus zeamais* (Coleoptera: Curculionidae) [24,25,26]. Therefore, we aimed to examine the repellent properties of EOs from *P. atriplicifolia*, *C. reticulata*, *D. ambrosioides*, *E. camaldulensis*, *C. citratus*, *M. longifolia*, *Salvia moorcroftiana* and *Azadirachta indica* plants against *Ae. aegypti*. Moreover, we identified the chemical composition of EOs that showed the highest repellent properties.

## 2. Results

### 2.1. Yield of Essential Oils

The aerial parts of *M. longifolia* and *P. atriplicifolia* were the richest in EO and yielded 1.33% and 1.21%, respectively, while the lowest amount (0.04%) of EO was produced from the bark of *A. indica* plants (Table 1).

### 2.2. Chemical Composition of Essential Oils

EOs of *P. atriplicifolia* (PA-EO), *C. reticulata* (II) fruit peels (CR-EO (II)), *M. longifolia* (ML-EO), *C. reticulata* (I) leaves (CR-EO (I)), and *D. ambrosioides* aerial parts at a fruiting stage (II) (DA-EO (II)) showed the highest repellent activity, therefore the chemical composition was investigated by GC–MS (Table 2). The most abundant compounds in the PA-EO were camphor (19.7%), eucalyptol (12.1%) and limonene (10.9%). The ML-EO was rich in carvone which constituted 82.6% of the EO. The major compounds of CR-EO (I) EO were sabinene (24.9%), limonene (23.1%) and linalool (15.6%) whereas CR-EO (II) was dominated by limonene that composes 92.7% of the EO. The main components in the EO extracted from DA-EO (II) contained 28.2% α-terpinene, 15.8% *p*-cymene and 38.8% *trans*-ascaridole (Table 2).

### 2.3. Repellency of Plant Essential Oils

In the screening bioassay, the numbers of female mosquito landings on the hand treated with different EOs or DEET were significantly lower (*p* < 0.001) than the negative control (solvent-treated hand). There were no mosquito landings on the hand treated with DEET, PA-EO and CR-EO (II) (Table 3).

**Figure 1:**
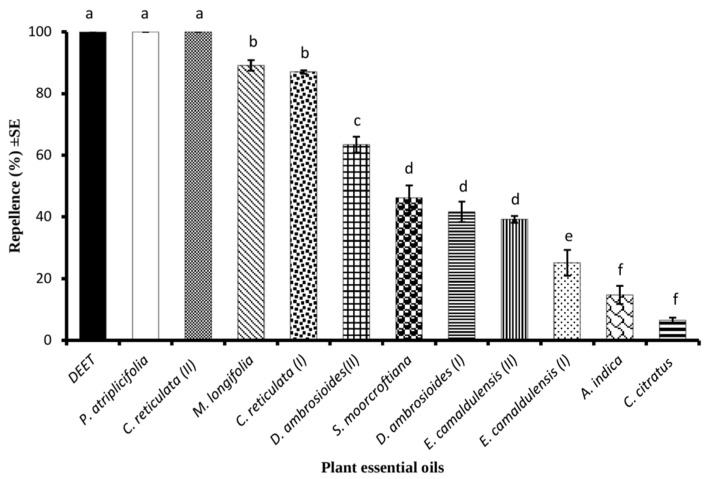
Screening of eleven plant essential oils as repellents against *Ae. aegypti* females at the tested dose of 33 μg/cm^2^. Bars with different letters are significantly different (*p* < 0.05) from each other. Error bars on each column present standard error where *n* = 5.

Out of eleven tested EOs, PA-EO and CA-EO (II) exhibited 100% of repellent effect which was similar (*p* > 0.05) to that of the positive control (DEET). The ML-EO and CR-EO (I) showed more than 85% of repellent effect whereas EO of *C. citratus* exhibited the lowest repellent effect of about 10% against *Ae. aegypti* females (Figure 1).

**Figure 2:**
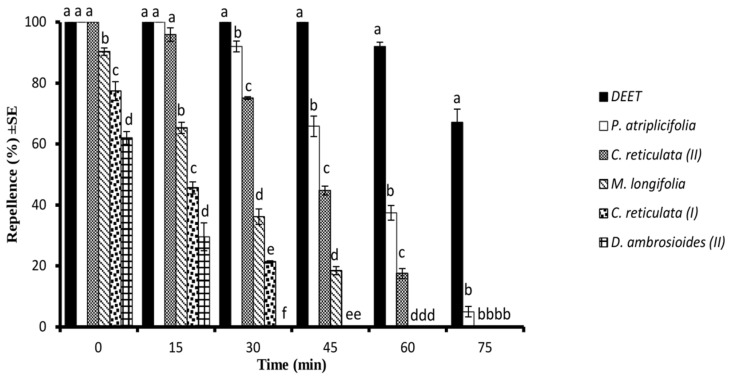
Time span mosquito repellency of different essential oils and DEET at the tested concentration of 33 μg/cm^2^ against *Ae. aegypti* females. Different letters on the bars show a significant difference (*p* < 0.05) among different test substances at a specific period. Error bars on each column represent standard error where *n* = 5.

In the time span bioassay, the PA-EO and CR-EO (II) at a concentration of 33 µg/cm^2^ exhibited 100% of repellent effect against *Ae. aegypti* until 15 min after the treatment which was similar (*p* > 0.05) to the repellent effect displayed by DEET (positive control). Both of these EOs were active for 60 min. The DA-EO (II) showed the shortest repellent effect and was active only up to 15 min of post-treatment (Figure 2).

**Figure 3:**
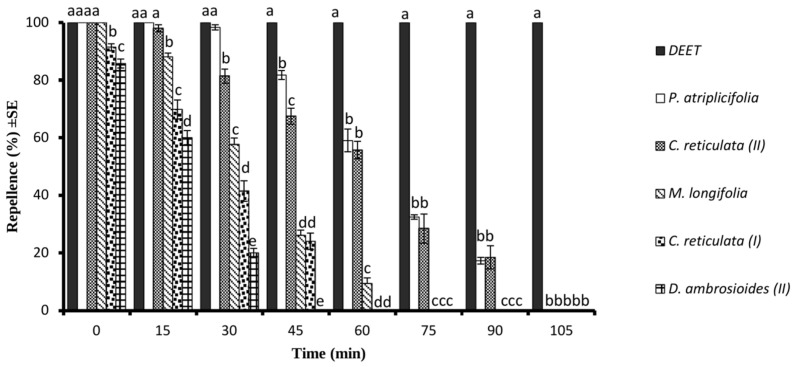
Time span of mosquito repellent effect of different essential oils and DEET at the tested dose of 165 μg/cm^2^ against *Ae. aegypti* females. Different letters on the bars show a significant difference (*p* < 0.05) among different test substances at a specific period. Error bars on each column represent standard error where *n* = 5.

At the tested concentration of 165 μg/cm^2^, PA-EO showed repellent effect similar to that of DEET (*p* > 0.05) for 30 min whereas *C. reticulata* CR-EO (II) showed repellent effect of 100% for 15 min. However, both PA-EO and CR-EO (II) exhibited an active time span of 90 min. The ML-EO exhibited repellent effect of 100% only for a few minutes of application; after that it decreased with time and showed repellent effect of about 12% after 60 min post-treatment. The DA-EO (II) possessed the lowest repellent effect which lasted up to 30 min (Figure 3).

**Figure 4:**
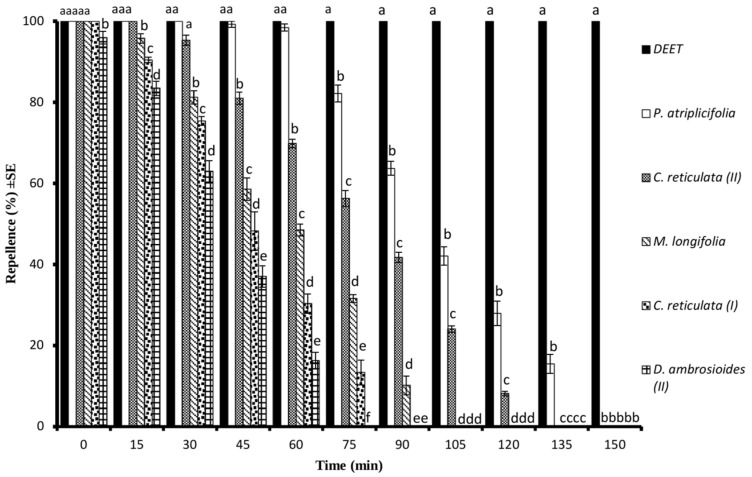
Time span mosquito repellency of different essential oils and DEET at the tested dose of 330 μg/cm^2^ against *Ae. aegypti* females. Different letters on the bars show a significant difference (*p* < 0.05) among different test substances at a specific period. Error bars on each column represent standard error where *n* = 5.

At the tested concentration of 330 μg/cm^2^, PA-EO showed complete protection similar to DEET (*p* > 0.05) for 60 min while CR-EO (II) exhibited complete protection for 30 min (Figure 4). The ML-EO showed complete protection immediately after application and the repellent effect lasted for 90 min. The highest time span of repellent effect, 135 min, was observed for PA-EO followed by the 120 min lasting repellent effect of CR-EO (II). The shortest repellence period of 60 min was observed for DA-EO (II) when tested at the highest dose of 330 μg/cm^2^ (Figure 4).

## 3. Discussion

Repellent formulations composed of EOs are finding increased popularity among consumers and are generally considered environmentally safe compared to synthetic repellents. Five out of 11 EOs, tested in the current study at 1% concentration, exhibited a repellent effect of more than 60% against females of *Ae. aegypti*. The most active five EOs were further studied to check their repelling longevity. In the time span bioassay, all five EOs showed repellent effect of varying degrees and different active time spans when tested at different concentrations. The PA-EO proved the most effective repellent among all plant EOs at all tested concentrations. Moreover, the repelling effect of PA-EO was comparable to DEET for an extended period of time.

To the best of our knowledge, the PA-EO has not been previously tested against any mosquito species, however, a few studies reported the bioactivity of *P. atriplicifolia* EO against other insect pests. For example, *P. atriplicifolia* along with gamma radiation showed antifeedant [21] as well as insecticidal activity against the adults of *T. castaneum* [27]. There are some studies reporting bioactivities of other plant species of the genus *Perovskia* towards insects. For example, *P. artemisioides* showed repellent properties against *Phthorimaea operculella* (Lepidoptera: Gelechiidae) [28] and acted as a toxic fumigant against *S. oryzae* and *T. castaneum* [29].

In the current study, chemical analysis revealed that camphor, eucalyptol, limonene and α-humulene were the most abundant compounds in the PA-EO. The higher proportion of these compounds in PA-EO could be the reason for its prolonged repellent effect towards *Ae. aegypti*. Previously, camphor and camphor containing a fraction of *Artemisia vulgaris* were reported to repel *Ae. aegypti* females at 140 µg/cm^2^ concentration [30]. A study from Sweden presented results similar to our findings, that camphor-rich *Tanacetum vulgare* (Asterales: Asteraceae) EO showed 90% repellent activity against ticks [31]. Another study reported camphor as a repellent and toxic fumigant against *Solenopsis invicta* [32]. Likewise, eucalyptol, the second most abundant compound of *P. atriplicifolia*, was reported as a moderate antifeedant and oviposition deterrent against *Ae. aegypti* [20]. Moreover, eucalyptol was also found to be a toxic fumigant compound against *An. sinensis* [33]. In another study, the EO of *Nepeta parnassica* (Lamiales: Lamiaceae), having eucalyptol as the main constituent, displayed a good repellency against *Ae. cretinus* and *Culex pipiens* (Diptera: Culicidae) for 3 h and 2 h, respectively [34].

The chemical profiles of EOs are influenced by many factors such as the time of sample collection, the soil type and the climate. The chemical composition of PA-EO reported in the current study is qualitatively different to that from a previous study from Pakistan [35] whereas qualitatively similar but quantitatively different in a study reported from Iran [36]. The chemical composition of CR-EO II was different to that from a study conducted in India that presented EO comprised of 50% limonene [37]. The composition of ML-EO was quite different in a recent study from Pakistan [38] but similar to a study from Greece [39]. The composition of *C. reticulata* leaves CR-EO (I) was different to CR-EO (II) studied here as well as in a previous study that reported the chemical composition of *C. reticulata* leaves [40]. The harvesting stage of *P. atriplicifolia* reported in the current study and that of Dabiri and Sefidkon [36] were similar, however, the climate, soil type, and other factors were different, so a small difference in the chemical composition was found in both studies. The differences between the EOs reported by Erdemgil et al. [35] and the current study could be due to different climatic conditions and harvesting times. The variations found in the chemical profiles of EOs might be due to the different geographical regions from where the plant samples were collected as well as the physical condition of the samples.

We showed that CR-EO (II) was the second most effective repellent after *P. atriplicifolia* against *Ae. aegypti*. At the highest tested concentration, CR-EO (II) was active for 120 min, however, complete protection similar to DEET was observed only for a period of 30 min. Previously, the EO extracted from the fruit peel of *C. reticulata* was reported as a repellent against *C. maculatus* [24] and *S. zeamais* [41] while ethanol extracted from *C. reticulata* showed repellent activity against *S. oryzae* [42]. Moreover, the larvicidal activity of *C. reticulata* peels against *Ae. aegypti* is also documented [43]. In the current study, the most abundant compound identified from CR-EO (II) was limonene (92.7%). A number of previous studies showed the bioactivities of limonene against a number of organisms. For example, Türkoğlu et al. reported that limonene applied to cotton fabric has been used to avoid mosquito bites [44]. Moreover, limonene was documented as a repellent against *Ae. aegypti* females [45]. In another study, the larvicidal activity of R-(+)-limonene was reported against larvae of *Ae. albopictus* [46]. Therefore, the presence of limonene in the CR-EO (II) could strongly contribute to the repellent activity against *Ae. aegypti*. However, the shorter protection span exhibited by CR-EO could be due to the high volatility of limonene.

ML-EO showed repellent activity for periods ranging from 45 to 90 min and the most abundant compound of this EO was carvone. The chemical composition and repellent effect of ML-EO determined in current experiments are different to those reported in previous studies carried out in Pakistan and Saudi Arabia. According to a study from Pakistan, *M. longifolia* EO showed repellent effect for 90 min against *Ae. aegypti* at the concentration of 330 μg/cm^2^ while its major identified compound was piperitone oxide [47]. The study from Saudi Arabia revealed that the *M. longifolia* EO was active against *C. pipiens* and showed repellent effect for more than 43 min but at higher concentration of 1000 μg/cm^2^ [48] which was about three times higher than that used in current study.

The CR-EO (I) showed moderate repellent activity that lasted for a shorter period of time. Although limonene was present as the main compound in CR-EO (I), the repellent activity of the sample was far lower than that of the same plant as CR-EO (II). This might be due to the synergetic effect of other compounds as well as the lower proportion of limonene in CR-EO (I). The DA-EO (II) exhibited the shortest protection period compared to the other four most active EOs investigated in the current study. This oil did not show complete protection immediately after application even at the highest tested concentration. Our results are similar to the repellent activity of *D. ambrosioides* EO against *Ae. aegypti* which was reported by a study from Argentina [49]. Interestingly, the main compounds in DA-EO (II) harvested at the fruiting stage were *trans*-ascaridole, α-terpinene and *p*-cymene whereas α-terpinene (41.4%), germacrene D (16.2%) and *p*-cymene (14.7%) were identified as the major compounds of DA-EO (I) extracted from same plant species harvested at vegetative stage [50]. Thus, the difference in the bioactivity of DA-EOs (I) and (II) could be explained by their major constituents.

In the current study, no skin irritation/allergic reaction was observed on the treated area of any volunteer’s hand. Moreover, the EOs did not have an unpleasant smell. In our previous study, skin irritation and an irritating odor of EOs were reported [51]. Therefore, on the basis of the previous observation, skin sensitivity tests need to be performed before recommending EOs for commercial use. Our study revealed that EOs extracted from the aerial parts of *P. atriplicifolia, M. longifolia* and fruit peel of *C. reticulata* showed potential to be used to formulate plant-based mosquito repellent against females of *Ae. aegypti*.

## 4. Materials and Methods

### 4.1. Insect Rearing

Larvae of *Ae. aegypti* were obtained from the Dengue Control Unit, Railway Hospital, Multan and established in the laboratory under controlled conditions (25 ± 2 °C, R.H 65 ± 5% and photoperiod 12L:12D) at the Department of Entomology, Faculty of Agricultural Sciences & Technology, Bahauddin Zakariya University, Multan, Pakistan. Larvae were placed in plastic containers filled with 1 L of tap water. A fish diet (crude protein, 28%, crude fat 3%, crude fiber 4% with 10% moisture) was used to feed the larvae. Pupae were collected daily in plastic cups containing tap water and transferred to PLEXIGLAS^®^ cages (30 × 30 × 30 cm) for adult emergence. Cotton soaked with 10% sucrose solution was placed in cages to feed the adults [19]. After 4–5 days, females were fed with blood using the immobilized pigeon method. A butter paper was placed on the inner side of the plastic jar filled with water and was kept in the adult cage as a substrate for oviposition. After oviposition, the butter paper with eggs was placed in the larval container filled with 1 L of tap water for mosquito hatching [9].

### 4.2. Collection of Plants

Different parts of plant species *Perovskia atriplicifolia*, *Citrus reticulata*, *Dysphania ambrosioides*, *Eucalyptus camaldulensis*, *Cymbopogon citratus*, *Mentha longifolia*, *Salvia moorcroftiana* and *Azadirachta indica* were collected from different areas of Pakistan. The parts of plants collected and their harvesting stage along with location coordinates and elevation are presented in Table 1. The identification of plant species was carried out by the plant taxonomist and the voucher specimens were submitted to the herbarium of the Department of Environmental Sciences, COMSATS University Islamabad, Abbottabad Campus, Abbottabad, Pakistan. The fresh plant material was either subjected to EO extraction on the same day of collection or stored in a freezer at −20 °C for 24–48 h until EO extraction.

### 4.3. Extraction of Essential Oils

The steam distillation method was used to extract the EO from the collected plant material as described in our previous publications [9,52]. A stainless-steel vessel (Liaqat Engineering Works, Faisalabad, Pakistan) was loaded with 2 kg of plant material and 2 L of distilled water. Water accumulated at the bottom of the steel vessel and had no direct contact with the plant material. The vessel was heated by an electric hot plate (Corning, NY, USA). Volatile compounds released from the plant materials along with steam were cooled down by using a condenser fitted on the head of the vessel and the distillate was collected in a separating funnel for 3 h. The collected distillate was extracted through liquid-liquid extraction using 210 mL (70 mL × 3) HPLC grade *n*-hexane (Daejung chemicals, Siheung, South Korea). The pooled hexane layers were dried by adding the anhydrous MgSO_4_ (Daejung chemicals, Siheung-si, South Korea)and filtered. The rotary evaporator (Buchi Labortechnik AG, Flawil, Switzerland) was used to evaporate the solvent at 25 °C under a vacuum. The obtained EO was weighed and the percentage yield of extracted EO was determined as described in our previous publication [53].

### 4.4. Chemical Analysis

Analysis of volatile compounds was carried out by using a HP 6890N gas chromatograph (GC) coupled with a HP 5973 mass spectrometer (MS) (Agilent Technologies Inc., Santa Clara, CA, USA). The GC was equipped with a DB-5 column (30 m length, 0.25 mm internal diameter, and 0.25 µm film coating comprised of 5% diphenyl and 95% dimethylpolysiloxane (Agilent, Santa Clara, CA, USA). The GC injector temperature was set at 235 °C while the GC oven temperature was maintained isothermally at 40 °C for 2 min, then increased at the rate of 4 °C/min up to 240 °C, and afterwards was kept isothermal for 8 min. Helium was used as a mobile phase with a constant flow of 1 mL/min. A diluted EO sample in *n*-hexane (Daejung chemicals, Siheung, South Korea) (500 ng/1 μL) was injected in a splitless mode set for 30 s. Electron ionization was performed at 70 eV where the ion source temperature was set constant at 180 °C. The mass spectra scan range was 30–400 amu. The total ion chromatogram peak area was used to find the percentage composition of each compound in an EO. EO components were identified by comparing their mass spectra with those present in the NIST-2008 MS library. Retention indices of separated compounds were determined relative to the retention times of a series of *n*-alkanes (C_9_–C_24_) (Merck, Darmstadt, Germany) analyzed at the same GC–MS parameters used for the EOs. Finally, the identified compounds were verified by injecting available standard compounds at the same parameters which were used for EOs analysis [9,53].

### 4.5. Repellency Bioassay

A human bait technique was used during the scotophase to test the repellence potential of EOs against *Ae. aegypti* females. Based on our previous experience, the 1%, 5%, and 10% solutions (10 mg/mL, 50 mg/mL, and 100 mg/mL,) of each EO and DEET were prepared using ethanol (Daejung chemicals, Siheung, South Korea) as a solvent. DEET (St. Louis, MO, USA) was used as a positive control. Twenty mated and blood-starved 4–5 days old female mosquitoes were released from the laboratory-reared colony in the experimental cage (30 × 30 × 30 cm). The hands of each subject were washed with scent-free liquid soap and allowed to dry for about 10 min before starting each test. Plastic gloves were used to cover each subject’s hand except for the 30 cm^2^ circular area on the dorsal side of the hand. An aliquot of 100 μL solution of test substance or pure solvent as a negative control was evenly applied on the exposed area of the hand and dried in air for three minutes before exposing the hand to mosquitoes. The hands of the subjects were exposed to the *Ae. aegypti* females in the experimental cage and mosquito landings, i.e., contact of the legs with the hand surface, were counted during the period of 5 min. The experiment was repeated randomly five times for both the test sample and the negative control. The human subjects (volunteers) were informed about the test procedure and consent was obtained before conducting repellency bioassays. The repellency percentage has been calculated by the formula presented by Azeem et al. [9]. Percentage repellency = [(M_c_ − M_t_)/M_c_] × 100 where M_c_ is the number of mosquito landings on the negative control (solvent) treated hand and M_t_ is the number of mosquito landings on the test substance treated hand.

### 4.6. Time Span Repellency Bioassay

Plant EOs that showed more than 60% repellency were further investigated to determine the maximum period of repellent activity. Time-span repellent bioassays were performed by following the same protocol as mentioned above in the repellency bioassay, except for the exposure of the same treated hand to the females of *Ae. aegypti* for 5 min after each 15 min time interval until the number of landings on control and treatment were the same. Time span bioassays were conducted by using test samples at the dosages of 33 μg/cm^2^, 165 μg/cm^2^ and 330 μg/cm^2^. The experiments were repeated five times and different females were employed for each replicate [17].

### 4.7. Statistical Analysis

Paired sample t-test was used to compare the number of landings of *Ae. aegypti* females on negative control and sample-treated hands. Statistical difference between the repellence of different EOs and DEET was analyzed by one-way ANOVA with post-hoc Bonferroni test. All the statistical tests were performed on Statistics 8 software (version 8.1, Tallahassee, FL, USA).

### 4.8. Ethical Approval

The Chairman of the Research Ethics and Biosafety Committee, Bahauddin Zakariya University, Multan, Pakistan provided the ethical approval.

## 5. Conclusions

The EO of *P. atriplicifolia* showed the best repellent activity of the EOs tested in our study. The complete protection by this EO from biting by females of *Ae. aegypti* lasted for more than 60 min and did not significantly differ from that of the gold-standard DEET. The EO of *P. atriplicifolia* could be further optimized, with the aim of developing an environmentally friendly and sustainable mosquito repellent formulation as an alternative to DEET.

## Figures and Tables

**Table 1:** Description of plants used and percent yield of the essential oils extracted by steam distillation process.

Voucher	Plant Name	Abbreviation	Family	Growth Stage	Plant Part Used	Plant Collection Site		Yield %
						Coordinates	Elevation (m)	
CUHA-225	*Dysphania ambrosioides*	*D. ambrosioides* (I)	Amaranthaceae	Pre-flowering	Aerial parts	34°12′11.0″ N 73°15′07.8″ E	1200	0.09
		*D. ambrosioides* (II)		Fruiting	Aerial parts	34°07′16.6″ N 73°19′54.73″ E	1300	0.23
CUHA-223	*Perovskia atriplicifolia*	*P. atriplicifolia*	Lamiaceae	Pre-flowering	Aerial parts	32°30′18.8″ N 69°45′00.3″ E	1950	1.21
CUHA-048	*Mentha longifolia*	*M. longifolia*		Pre-flowering	Aerial parts	34°07′20.5″ N 73°19′58.3″ E	1300	1.33
CUHA-176	*Salvia moorcroftiana*	*S. moorcroftiana*		Flowering	Flowers	34°12′39.1″ N 73°18′38.4″ E	1680	0.06
CUHA-227	*Azadirachta indica*	*A. indica*	Meliaceae	Flowering	Bark	30°16′06.6″ N 71°30′05.8″ E	120	0.04
CUHA-226	*Eucalyptus camaldulensis*	*E. camaldulensis* (I)	Myrtaceae	Flowering	Leaves	34°11′56.7″ N 73°14′37.1″ E	1200	0.51
		*E. camaldulensis* (II)			Flower buds	34°11′56.7″ N 73°14′37.1″ E	1200	0.32
CUHA-228	*Cymbopogon citratus*	*C. citratus*	Poaceae	Pre-flowering	Aerial parts	31°28′19.0″ N 73°12′49.1″ E	180	0.31
CUHA-224	*Citrus reticulata*	*C. reticulata* (I)	Rutaceae	Mature fruit stage	Leaves	30°59′20.24″ N 72°53′21.84″ E	170	0.15
		*C. reticulata* (II)			Fruit peels	30°59′20.24″ N 72°53′21.84″ E	170	0.29

**Table 2:** Chemical composition of plant essential oils based on total ion chromatogram of GC-MS.

Compound Name	RI ^‡^	*PA*	*ML*	*CR* (I)	*CR* (II)	*DA* (II)	IM *
α-Thujene	924	0.5		0.4			MS, RI
α-Pinene	928	3.8	0.1	1.5	0.5	0.3	Std
Camphene	943	3.8		0.1			Std
Sabinene	969	0.3		24.9	1.6		MS, RI
β-Pinene	972	2.3	0.1	1.8	0.2	0.6	Std
6-Methyl-5-heptene-2-one	984			0.6			MS, RI
β-Myrcene	988	0.7	0.1	2.9	2.5		MS, RI
3-Octanol	993		0.2				MS, RI
α-Phellandrene	1002	0.1			0.1		MS, RI
3-Carene	1008	0.4					MS, RI
α-Terpinene	1015	0.3		1.1	0.1	28.2	MS, RI
*p*-Cymene	1023	2.0		0.6		15.8	MS, RI
Limonene	1028	10.9	3.9	23.1	92.7	0.2	Std
Eucalyptol	1031	12.1	1.9			0.8	Std
*cis*-β-Ocimene	1036	0.2	0.1	1.1	0.3		Std
*trans*-β-Ocimene	1047	0.1		4.9			Std
γ-Terpinene	1058	2.3		2.1	0.2	0.5	MS, RI
*cis*-Sabinene hydrate	1066	0.3	0.1	0.2			MS, RI
Terpinolene	1088	0.3		0.6	0.1		MS, RI
Dehydro-*p*-cymene	1091					0.3	MS, RI
*trans*-Sabinene hydrate	1098	0.2					MS, RI
Linalool	1100	0.3	0.2	15.6	0.2		Std
*cis-p*-Menth-2-ene-1-ol	1121			0.2		0.2	MS, RI
Phenylacetonitrile	1141			1.3			MS, RI
*trans-p*-Menth-2-ene-1-ol	1142			0.2		1.6	MS, RI
Camphor	1147	19.7					Std
β-Citronellal	1152			1.5	0.1		MS, RI
Borneol	1167	2.5	3.2				Std
4-Terpineol	1179	0.3	0.2	5.1	0.2		Std
α-Terpineol	1192	0.4	0.2	1.6	0.1		Std
1,6-Dihydrocarveol	1195		0.6				MS, RI
*cis*-Dihydrocarvone	1196		1.4				MS, RI
*trans*-Carveol	1219		0.3				MS, RI
*cis*-Geraniol	1228			0.4			MS, RI
*cis*-Carveol	1232		0.2				MS, RI
β-Citral	1241			1.7			MS, RI
Carvone	1247		82.6				Std
*cis*-Ascaridole	1247					6.3	MS, RI
*trans*-Geraniol	1254			0.2			MS, RI
Piperitone	1256		0.2			1.5	MS, RI
*cis*-Carvenone oxide	1264		0.1			3.6	MS, RI
α-Citral	1270			2.2			MS, RI
*trans*-Carvone oxide	1278		0.2				MS, RI
Bornyl acetate	1288	4.2					Std
*trans*-Ascaridole	1312					38.8	MS, RI
α-Terpinyl acetate	1352	1.6					MS, RI
α-Copaene	1381	0.4					Std
α-Gurjunene	1416	0.2					MS, RI
*trans*-β-Caryophyllene	1427	5.5	1.0	1.4	0.2		MS, RI
*trans*-α-Bergamotene	1440				0.3		MS, RI
α-Humulene	1461	6.3		0.2			MS, RI
*trans*-β-Farnesene	1458		0.2	0.2			MS, RI
allo-Aromadendrene	1468	1.1					MS, RI
Germacrene D	1487		0.3				MS, RI
Viridiflorene	1502	2.2					MS, RI
*trans*-α-Farnesene	1509			0.2			MS, RI
β-Bisabolene	1513				0.2		MS, RI
*cis*-Lachnophyllum ester	1514			0.5			MS, RI
γ-Cadinene	1520	0.6					MS, RI
δ-Cadinene	1529	1.4					MS, RI
Caryophyllene oxide	1588	1.6	1.0			0.1	MS, RI
Ledol	1600	8.1					MS, RI
δ-Cadinol	1647	0.8					MS, RI
Monoterpenes		28.0	4.3	65.0	98.4	45.8	
Oxygenated monoterpenes		41.6	91.3	29.0	0.6	52.7	
Sesquiterpenes		17.7	1.6	1.9	0.6	0.0	
Oxygenated sesquiterpenes		10.5	1.0			0.1	
Others			0.2	2.4			
Total Identified		97.8	98.4	98.2	99.5	98.6	

*PA* = *Perovskia atriplicifolia*; *ML* = *Mentha longifolia*; *CR*(I) = *Citrus reticulata* (fruit peels); *CR*(II) = *C. reticulata* (leaves); *DA*(II) = *Dysphania ambrosioides* (fruiting aerial parts). * In the identification method IM; MS = identification based on mass spectrum comparison with NIST-2008 library, RI = identification based on comparison of retention index with published data and Std = identification of compounds was made by comparing mass spectrum, retention index with published data as well as through the injection of standard compounds. ^‡^ Compounds listed are in order of elution from a DB-5 GC column. The retention index (RI) of a separated compound was calculated relative to C_9_–C_26_ retention time on the same parameter used for EOs analysis. The data shown in the table is the percentage compositions of different EOs where the values < 0.5% are approximate.

**Table 3:** Mosquito landings on human hand treated with different essential oils, DEET and negative control.

Tested Substances	Average Number of Landings on Control (Negative)	Average Number of Landings on the Test Substance	*p* Value	df, t
*DEET*	44.5 ± 1.76	0.00 ± 0.00	<0.001	4, 25.21
*P. atriplicifolia*	35.33 ± 2.10	0.00 ± 0.00	<0.001	4, 16.76
*C. reticulata* (II)	30.16 ± 1.13	0.00 ± 0.00	<0.001	4, 26.51
*M. longifolia*	34.0 ± 0.93	3.16 ± 0.30	<0.001	4, 29.47
*C. reticulata* (I)	35.00 ± 1.3	4.50 ± 0.22	<0.001	4, 23.28
*D. ambrosioides* (II)	28.66 ± 0.84	9.66 ± 0.33	<0.001	4, 19.67
*S. moorcroftiana*	37.16 ± 1.92	19.16 ± 0.60	<0.001	4, 10.63
*D. ambrosioides* (I)	38.00 ± 1.90	22.00 ± 1.18	<0.001	4, 12.92
*E. camaldulensis* (II)	37.00 ± 1.00	22.66 ± 0.84	<0.001	4, 67.99
*E. camaldulensis* (I)	40.83 ± 1.30	30.33 ± 1.08	<0.001	4, 8.53
*A. indica*	39.66 ± 2.52	34.16 ± 2.65	<0.001	4, 9.77
*C. citratus*	46.00 ± 1.00	42.5 ± 1.17	<0.001	4, 8.17

## Data Availability

The data presented in this study are available on request from the corresponding author.

## Sample Availability

Samples of the compounds are available from the authors.

## References

[1] WRBU Walter Reed Biosystematics Unit (WRBU) Database. The USA. www.wrbu.si.edu.

[2] Iwamura T., Guzman-Holst A., Murray K.A. (2020). Accelerating invasion potential of disease vector *Aedes aegypti* under climate change. Nat. Commun..

[3] Debboun M., Frances S.P., Strickman D. (2006). Insect Repellents: Principles, Methods, and Uses.

[4] Mbuba E., Odufuwa O., Tenywa F., Philipo R., Tambwe M., Swai J.K., Moore J., Moore S. (2021). Single blinded semi-field evaluation of MAÏA^®^ topical repellent ointment compared to unformulated 20% DEET against *Anopheles gambiae*, *Anopheles arabiensis* and *Aedes aegypti* in Tanzania. Malar. J..

[5] Gryboski J., Weinstein D., Ordway N.K. (1961). Toxic encephalopathy is apparently related to the use of an insect repellent. N. Engl. J. Med..

[6] Lamberg S.I., Mulrennan J.A. (1969). Bullous Reaction to Diethyl Toluamide (DEET): Resembling a Blistering Insect Eruption. Arch. Dermatol..

[7] Windheuser J.J., Haslamx J.L., Caldwell L., Shaffer R.D. (1982). The use of N, N-diethyl-m-toluamide to enhance dermal and transdermal delivery of drugs. J. Pharm. Sci..

[8] Ali A., Tabanca N., Demirci B., Raman V., Budel J.M., Baser K., Khan I.A. (2020). Insecticidal and biting deterrent activities of *Magnolia grandiflora* essential oils and selected pure compounds against *Aedes aegypti*. Molecules.

[9] Azeem M., Zaman T., Tahir M., Haris A., Iqbal Z., Binyameen M., Nazir A., Shad S.A., Majeed S., Mozūraitis R. (2019). Chemical composition and repellent activity of native plants essential oils against dengue mosquito, *Aedes aegypti*. Ind. Crops Prod..

[10] Soonwera M. (2015). Efficacy of essential oils from Citrus plants against mosquito vectors *Aedes aegypti* (Linn.) and *Culex quinquefasciatus* (Say). J. Agric. Technol..

[11] Sritabutra D., Soonwera M., Waltanachanobon S., Poungjai S. (2011). Evaluation of herbal essential oil as repellents against *Aedes aegypti* (L.) and *Anopheles dirus* Peyton & Harrison. Asian Pac. J. Trop. Biomed..

[12] Phasomkusolsil S., Soonwera M. (2010). Insect repellent activity of medicinal plant oils against *Aedes aegypti* (Linn.), *Anopheles minimus* (Theobald) and *Culex quinquefasciatus* Say based on protection time and biting rate. Southeast Asian J. Trop. Med. Public Health.

[13] Tunón H., Thorsell W., Mikiver A., Malander I. (2006). Arthropod repellency, especially tick (*Ixodes ricinus*), exerted by extract from *Artemisia abrotanum* and essential oil from flowers of *Dianthus caryophyllum*. Fitoterapia.

[14] Kumar S., Wahab N., Warikoo R. (2011). Bioefficacy of *Mentha piperita* essential oil against dengue fever mosquito *Aedes aegypti* L.. Asian Pac. J. Trop. Biomed..

[15] Reichert W., Ejercito J., Guda T., Dong X., Wu Q., Ray A., Simon J.E. (2019). Repellency assessment of *Nepeta cataria* essential oils and isolated nepetalactones on *Aedes aegypti*. Sci. Rep..

[16] Venkatachalam M., Jebanesan A. (2001). Repellent activity of *Ferronia elephantum* Corr. (Rutaceae) leaf extract against Aedes aegypti (L.). Bioresour. Technol..

[17] Haris A., Azeem M., Binyameen M. (2022). Mosquito Repellent Potential of *Carpesium abrotanoides* Essential Oil and Its Main Components Against a Dengue Vector, *Aedes aegypti* (Diptera: Culicidae). J. Med. Entomol..

[18] Choi W.-S., Park B.-S., Ku S.-K., Lee S.-E. (2002). Repellent activities of essential oils and monoterpenes against *Culex pipiens* pallens. J. Am. Mosq. Control Assoc..

[19] Nararak J., Sathantriphop S., Kongmee M., Mahiou-Leddet V., Ollivier E., Manguin S., Chareonviriyaphap T. (2019). Excito-repellent activity of β-caryophyllene oxide against *Aedes aegypti* and *Anopheles minimus*. Acta Trop..

[20] Klocke J.A., Darlington M.V., Balandrin M.F. (1987). 1, 8-Cineole (Eucalyptol), a mosquito feeding and ovipositional repellent from volatile oil of *Hemizonia fitchii* (Asteraceae). J. Chem. Ecol..

[21] Ahmadi M., Moharramipour S., Abd Alla A. (2015). Antifeedant effect of gamma radiation and *Perovskia atriplicifolia* essential oil combination against *Tribolium castaneum* (Coleoptera: Tenebrionidae). J. Crop Prot..

[22] Kathuria V., Kaushik N. (2005). Feeding inhibition of *Helicoverpa armigera* (Hübner) by *Eucalyptus camaldulensis* and *Tylophora indica* extracts. Insect Sci..

[23] Negahban M., Moharramipour S. (2007). Fumigant toxicity of *Eucalyptus intertexta*, *Eucalyptus sargentii* and *Eucalyptus camaldulensis* against stored-product beetles. J. Appl. Entomol..

[24] Saeidi M., Moharramipour S., Sefidkon F., Aghajanzadeh S. (2011). Insecticidal and repellent activities of *Citrus reticulata*, *Citrus limon* and *Citrus aurantium* essential oils on *Callosobruchus maculatus*. Integr. Prot. Stored Prod. IOBC/WPRS Bull..

[25] Saeidi M., Moharramipour S. (2013). Insecticidal and repellent activities of *Artemisia khorassanica, Rosmarinus officinalis* and *Mentha longifolia* essential oils on *Tribolium confusum*. J. Crop Prot..

[26] Langsi D., Nukenine E., Fokunang C., Suh C., Goudoungou W. (2017). Potentials of essential oils of *Chenopodium ambrosioides* L. and *Cupressus sempervirens* L. against stored maize pest, *Sitophilus zeamais* Motschulsky. J. Entomol. Zool. Stud..

[27] Ahmadi M., Abdalla A.M.M., Moharramipour S. (2013). Combination of gamma radiation and essential oils from medicinal plants in managing *Tribolium castaneum* contamination of stored products. Appl. Radiat. Isot..

[28] Sadeghi Z., Alizadeh Z., Khorrami F., Norouzi S., Moridi Farimani M. (2021). Insecticidal activity of the essential oil of *Perovskia artemisioides* Boiss. Nat. Prod. Res..

[29] Arabi F., Moharramipour S., Sefidkon F. (2008). Chemical composition and insecticidal activity of essential oil from *Perovskia abrotanoides* (Lamiaceae) against *Sitophilus oryzae* (Coleoptera: Curculionidae) and *Tribolium castaneum* (Coleoptera: Tenebrionidae). Int. J. Trop. Insect Sci..

[30] Hwang Y.-S., Wu K.-H., Kumamoto J., Axelrod H., Mulla M.S. (1985). Isolation and identification of mosquito repellents in *Artemisia vulgaris*. J. Chem. Ecol..

[31] Pålsson K., Jaenson T.G., Bæckström P., Borg-Karlson A.-K. (2008). Tick repellent substances in the essential oil of *Tanacetum vulgare*. J. Med. Entomol..

[32] Fu J., Tang L., Li W., Wang K., Cheng D., Zhang Z. (2015). Fumigant toxicity and repellence activity of camphor essential oil from *Cinnamonum camphora* Siebold against *Solenopsis invicta* workers (Hymenoptera: Formicidae). J. Insect Sci..

[33] Luo D.-Y., Yan Z.-T., Che L.-R., Zhu J.J., Chen B. (2022). Repellency and insecticidal activity of seven Mugwort (*Artemisia argyi)* essential oils against the malaria vector *Anopheles sinensis*. Sci. Rep..

[34] Gkinis G., Michaelakis A., Koliopoulos G., Ioannou E., Tzakou O., Roussis V. (2014). Evaluation of the repellent effects of *Nepeta parnassica* extract, essential oil, and its major nepetalactone metabolite against mosquitoes. Parasitol. Res..

[35] Erdemgil F.Z., Ilhan S., Korkmaz F., Kaplan C., Mercangöz A., Arfan M., Ahmad S. (2007). Chemical composition and biological activity of the essential oil of *Perovskia atriplicifolia*. from Pakistan. Pharm. Biol..

[36] Dabiri M., Sefidkon F. (2001). Analysis of the essential oil from aerial parts of *Perovskia atriplicifolia* Benth. at different stages of plant growth. Flavour Fragr. J..

[37] Goyal L., Kaushal S. (2018). Evaluation of chemical composition and antioxidant potential of essential oil from *Citrus reticulata* fruit peels. Adv. Res..

[38] Abbas M.G., Haris A., Binyameen M., Nazir A., Mozūratis R., Azeem M. (2022). Chemical composition, larvicidal and repellent activities of wild plant essential oils against *Aedes aegypti*. Biology.

[39] Koliopoulos G., Pitarokili D., Kioulos E., Michaelakis A., Tzakou O. (2010). Chemical composition and larvicidal evaluation of *Mentha*, *Salvia*, and *Melissa* essential oils against the West Nile virus mosquito *Culex pipiens*. Parasitol. Res..

[40] Lawal O.A., Ogunwande I.A., Owolabi M.S., Giwa-Ajeniya A.O., Kasali A.A., Abudu F.A., Sanni A.A., Opoku A.R. (2014). Comparative analysis of essential oils of *Citrus aurantifolia* Swingle and *Citrus reticulata* Blanco, from two different localities of Lagos State, Nigeria. Am. J. Essent. Oils Nat. Prod..

[41] Fouad H.A., da Camara C.A. (2017). Chemical composition and bioactivity of peel oils from *Citrus aurantiifolia* and *Citrus reticulata* and enantiomers of their major constituent against *Sitophilus zeamais* (Coleoptera: Curculionidae). J. Stored Prod. Res..

[42] Akhtar M., Arshad M., Raza A.B.M., Chaudhary M.I., Iram N., Akhtar N., Mahmood T. (2013). Repellent effects of certain plant extracts against rice weevil, *Sitophilus oryzae* L.(Coleoptera: Curculionidae). Int. J. Agric. Appl. Sci..

[43] Sutthanont N., Choochote W., Tuetun B., Junkum A., Jitpakdi A., Chaithong U., Riyong D., Pitasawat B. (2010). Chemical composition and larvicidal activity of edible plant-derived essential oils against the pyrethroid-susceptible and -resistant strains of *Aedes aegypti* (Diptera: Culicidae). J. Vector Ecol..

[44] Türkoğlu G.C., Sarıışık A.M., Erkan G., Yıkılmaz M.S., Kontart O. (2020). Micro-and nano-encapsulation of limonene and permethrin for mosquito repellent finishing of cotton textiles. Iran. Polym. J..

[45] Nematollahi N., Ross P.A., Hoffmann A.A., Kolev S.D., Steinemann A. (2021). Limonene Emissions: Do Different Types Have Different Biological Effects?. Int. J. Environ. Res. Public Health.

[46] Giatropoulos A., Papachristos D.P., Kimbaris A., Koliopoulos G., Polissiou M.G., Emmanouel N., Michaelakis A. (2012). Evaluation of bioefficacy of three Citrus essential oils against the dengue vector *Aedes albopictus* (Diptera: Culicidae) in correlation to their components enantiomeric distribution. Parasitol. Res..

[47] Iqbal S., Khan F.A., Haris A., Mozuratis R., Binyameen M., Azeem M. (2023). Essential oils of four wild plants inhibit the blood seeking behaviour of female *Aedes aegypti*. Exp. Parasitol..

[48] Al-Sarar A. (2014). Chemical Composition, Adulticidal and Repellent Activity of Essential Oils From *Mentha longifolia* L. and *Lavandula dentata* L. against *Culex pipiens* L.. J. Plant Prot. Pathol..

[49] Gillij Y., Gleiser R., Zygadlo J. (2008). Mosquito repellent activity of essential oils of aromatic plants growing in Argentina. Bioresour. Technol..

[50] Azeem M., Zaman T., Abbasi A.M., Abid M., Mozūratis R., Alwahibi M.S., Elshikh M.S. (2022). Pesticidal potential of some wild plant essential oils against grain pests *Tribolium castaneum* (Herbst, 1797) and *Aspergillus flavus* (Link, 1809). Arab. J. Chem..

[51] Barnard D.R. (1999). Repellency of essential oils to mosquitoes (Diptera: Culicidae). J. Med. Entomol..

[52] Kumar S., Ahmad R., Saeed S., Azeem M., Mozūraitis R., Borg-Karlson A.-K., Zhu G. (2022). Chemical composition of fresh leaves headspace aroma and essential oils of four Coriander cultivars. Front. Plant Sci..

[53] Azeem M., Iqbal Z., Emami S.N., Nordlander G., Nordenhem H., Mozūratis R., El-Seedi H.R., Borg-Karlson A.K. (2020). Chemical composition and antifeedant activity of some aromatic plants against pine weevil (*Hylobius abietis*). Ann. Appl. Biol..

